# Metabolic Engineering Strategies for Improved Lipid Production and Cellular Physiological Responses in Yeast *Saccharomyces cerevisiae*

**DOI:** 10.3390/jof8050427

**Published:** 2022-04-21

**Authors:** Wei Jiang, Chao Li, Yanjun Li, Huadong Peng

**Affiliations:** 1 Department of Chemical and Biological Engineering, Monash University, Clayton, VIC 3800, Australia; 2 The Novo Nordisk Foundation Center for Biosustainability, Technical University of Denmark, 2800 Kongens Lyngby, Denmark; jiangw@biosustain.dtu.dk; 3 State Key Laboratory of Bioreactor Engineering, East China University of Science and Technology, Shanghai 200234, China; lichao@ecust.edu.cn; 4 College of Biotechnology, Tianjin University of Science and Technology, Tianjin 300457, China; yjli@tust.eud.cn; 5 Key Laboratory of Industrial Fermentation Microbiology, Ministry of Education, Tianjin University of Science and Technology, Tianjin 300457, China

**Keywords:** metabolic engineering, synthetic biology, yeast, triacylglycerol, cellular physiology, fatty acid

## Abstract

Microbial lipids have been a hot topic in the field of metabolic engineering and synthetic biology due to their increased market and important applications in biofuels, oleochemicals, cosmetics, etc. This review first compares the popular hosts for lipid production and explains the four modules for lipid synthesis in yeast, including the fatty acid biosynthesis module, lipid accumulation module, lipid sequestration module, and fatty acid modification module. This is followed by a summary of metabolic engineering strategies that could be used for enhancing each module for lipid production. In addition, the efforts being invested in improving the production of value-added fatty acids in engineered yeast, such as cyclopropane fatty acid, ricinoleic acid, gamma linoleic acid, EPA, and DHA, are included. A discussion is further made on the potential relationships between lipid pathway engineering and consequential changes in cellular physiological properties, such as cell membrane integrity, intracellular reactive oxygen species level, and mitochondrial membrane potential. Finally, with the rapid development of synthetic biology tools, such as CRISPR genome editing tools and machine learning models, this review proposes some future trends that could be employed to engineer yeast with enhanced intracellular lipid production while not compromising much of its cellular health.

## 1. Introduction

Microbial lipids are valuable as sustainable feedstock alternatives to crude oil for the production of chemicals and fuels. Microbes that have been metabolically engineered to improve productivity can produce large quantities of renewable chemicals from simple, readily available, inexpensive starting materials [1,2]. There are several advantages to using the sustainable approach of metabolically engineered microorganisms to produce biofuel or chemicals: (1) it can avoid competition with food-derived sugars by supplying non-food carbon feedstocks, such as cellulosic sugars; (2) it has the flexibility in fatty acid (FA) type produced that are suited to various industrial applications, such as biofuels, surfactants, and lubricants [3,4]; (3) production can be scaled up to satisfy growing worldwide oleochemicals demand for renewable non-food sources of FA [4]; and (4) the approach reduces deforestation and greenhouse gas emissions caused by widespread land clearing, which is required for plant oil production (such as oil palm) [5,6].

Research into the improvement in productivity of microbial lipids through metabolic engineering has been hot, developed fast, and progressed recently. It is necessary to review the status and achievements of lipid metabolism that could guide the design–build–test–learn (DBTL) cycle of microbial cell factories for lipid production. This manuscript aims to review the mechanisms, progress, and challenges of *de novo* lipid production in engineered yeast *S. cerevisiae* due to its advantages of being a very well-studied organism with a wide range of strains and excellent genetic tools available. It stores lipids as stable droplets and has a proven track record in industrial applications. Moreover, successful metabolic engineering strategies developed in *S. cerevisiae* may be easily transferred to other useful industrial organisms, such as the oleaginous yeast *Yarrowia lipolytica.*

### 1.1. Host Microorganisms

Fatty acid synthesis is one of the ubiquitous pathways in organisms, including bacteria, fungi, algae, plants, etc. Most bacteria, such as *E. coli* and *Archaea,* synthesize FAs and use them as membrane components, while storing energy in the form of polyhydroxyalkanoates instead of TAG in lipid droplets (LDs), as seen in eukaryotes [7,8]. Nevertheless, few bacteria have the ability to synthesize TAG. The short generation time and industrial track record of *E. coli* attract attention to using it as a chassis for lipid engineering. For example, the *E. coli* MG1655 dgkA mutant strain achieved a TAG yield (9%, *w/w*) by overexpressing *fadD* and two copies of *atfA*, which encodes wax synthase (WS)/DGAT from *Acinetobacter baylyi* [8,9]. Additionally, a very high yield of medium-chain FAs (3.8 g/L) were reached via the reverse beta-oxidation cycle [10]. However, the lipid yield is still insufficient to reach levels that meet commercialization requirements.

Due to the advantages of having high cellular lipid content (>60%, dry cell weight, DCW) and the ability to photosynthesize, microalgae have long been designated for lipid production, including the well-studied model microalgae from among Chlorophyta, Bacillariophyceae [11]. However, there are also some obstacles, such as low biomass concentrations, high dewatering cost, high water demand, and high oil recovery costs, that limit the development of microalgae as a lipid production cell factory [12,13]. Additionally, the cultivation of microalgae is also biotechnologically challenging due to the fact that photosynthesis requires illumination, CO_2_ (limited by CO_2_ content of air), and moderate temperatures (limitation of geographical development). In addition, algae culture in open ponds is difficult because there are predators, such as protozoa, viruses, and diseases from other algae or bacteria [14,15].

In oil-producing plants, such as *Brassica napus*, *Nicotiana tabacum*, and *palm*, several effective metabolic engineering approaches have been undertaken, including the overexpression of acyltransferase genes obtained from model plants, such as *Arabidopsis thaliana* in *tobacco* and *Brassica napus*, to enhance TAG production in seed oils, fruit, pollen grains, or leaves of the transformed hosts [16,17,18]. However, plant oil production can result in deforestation because of the widespread land clearing required to establish new oil crops, which may make the process environmentally unsustainable [19], especially when the product is for non-food purposes. Furthermore, it takes many years or decades to establish genetically modified organism (GMO) crop plants, such as oil-bearing crambe or brassicas, mainly due to the time taken for official approval and establishing the production and value chain [20].

Around 60 species of oleaginous yeasts can produce high yields of TAG, more than 20% dry cell weight (DCW), such as *Yarrowia lipolytica*, *Cutaneotrichosporon oleaginosus, Lipomyces starkeyi,* and *Rhodosporidium toruloides* [21]. These oleaginous yeasts have naturally evolved with high flux pathways for fatty acids in the form of neutral lipids, which can be converted into a variety of drop-in fuels and oleochemicals [22]. Among those oleaginous yeasts, much more progress has been achieved in increasing lipid production in *Y. lipolytica* because of the well-developed genetic engineering tools; for example, Xu et al. (2017) reported the highest reported to date lipid titer that could reach 115 g/L in a semicontinuous mode using acetic acid as substrates [23].

While the lipid content of most yeasts/fungi is normally low, they are important sources of metabolites such as for nutritional and pharmaceutical applications, especially polyunsaturated FAs (PUFAs), eicosapentaenoic acid (20:5 ω-3; EPA), docosahexaenoic acid (22:6 ω-3; DHA), and γ-linolenic acid (18:3 ω-6; GLA) [14,15,24,25]. Baker’s yeast *Saccharomyces cerevisiae*, despite not being classified as oleaginous, has many advantages that could be exploited when being used for metabolic engineering for lipid production and has thus been widely studied. For example, it is highly tractable and is available in a wide range of genetic backgrounds; there are abundant molecular tools, large assortment of commercially available deletion strains, short generation time, easy to culture, and proven track record in various industry applications [26].

As shown in Table 1, each popular host is not ideal for lipid production, containing both pros and cons. While all four main lipid production sources are the subjects of intensive research, this review mainly focuses on yeast *S. cerevisiae* as the host organism owing to some of its attractive advantages, namely its capability of storing lipids stably and its proven track record in industrial applications.

### 1.2. Lipid Synthesis in Yeast

Fatty acids (FAs) are hydrocarbon derivatives; they are carboxylic acids with hydrocarbon chains ranging from 4 to 36 carbons long (C4 to C36). In some FAs, this chain is unbranched and fully saturated (without double bonds), such as palmitic acid (C16:0) and stearic acid (C18:0); in others, the chain contains one or more double bonds, such as palmitoleic acid (C16:1, △9), oleic acid (C18:1, △9), and linoleic acid (C18:2, △9, 12) [28]. Cellular lipids can be classified into two major categories, namely energy storage lipids (neutral) and membrane lipids (polar). Triacylglycerols (TAGs) are the simplest neutral lipids constructed from FAs, also referred to as triglycerides, fats, or neutral fats, which contain three FA molecules esterified to three hydroxyl groups of glycerol. Phospholipids (PLs) and sterols are major structural elements of biological membranes. Membrane lipids are amphipathic, with one end of the lipid molecule being hydrophobic and the other end being hydrophilic. Among membrane lipids, PLs use a polar head group to join the hydrophobic moiety through the phosphodiester linkage [14,28].

**Figure 1:**
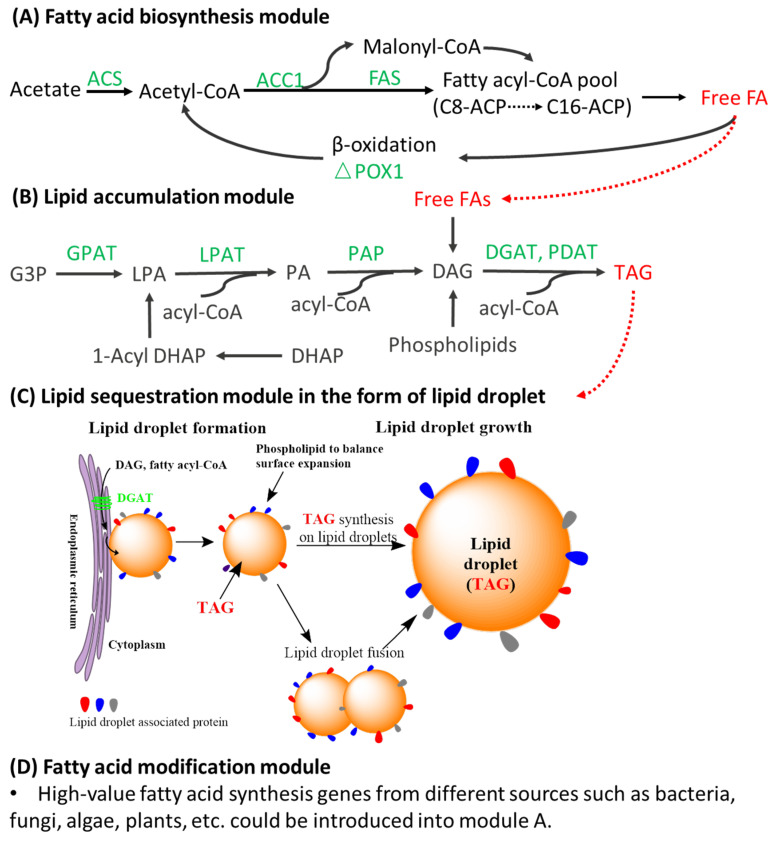
Four modules of lipid biosynthesis in yeast, including (**A**) fatty acid biosynthesis module, (**B**) lipid accumulation module, (**C**) lipid sequestration module in the form of lipid droplet, endoplasmic reticulum budding model of lipid droplet formation and expansion (redrawn with modifications from [29], and (**D**) fatty acid modification module.

From carbon source to lipid production, there are four major modules in yeast: FA synthesis, FA modification, lipid accumulation, and sequestration, as shown in Figure 1. FAs represent a suitable storage compound for energy and carbon because the degradation of FAs yields a high amount of adenosine triphosphate (ATP) and reducing equivalents [7]. FA modification may occur on the acyl chain while it is attached to Coenzyme A (CoA) or for some unusual FAs, such as cyclopropane and ricinoleic acid; it may occur when the FA is attached to another substrate, which would be discussed in Section 2.4. In multicellular organisms, unicellular eukaryotes, and some prokaryotes, FAs are stored in the form of TAG or wax esters, a process described herein as FA accumulation. The surplus neutral lipids, such as TAG and sterol ester (SE), can then be sequestrated into lipid droplets (LDs). LD organelles are a stable reservoir of stored lipids within the cell. LDs provide the most efficient form of energy storage due to the package of highly reduced hydrophobic lipids, such as TAG, in a phase without water. LDs can also provide building blocks for cellular membranes or substrates for energy metabolism [29,30].

FA synthesis requires the participation of a three-carbon intermediate, malonyl-CoA, which is formed from acetyl-CoA and bicarbonate. The formation of malonyl-CoA from acetyl-CoA is an irreversible process catalyzed by acetyl-CoA carboxylase (ACC) (Figure 1), which contains a biotin prosthetic group covalently bound in an amide linkage to the ε-amino group of a lysine residue in one of the three polypeptides or domains of the enzyme molecule. The carboxyl group, derived from bicarbonate (HCO_3_^−^), first transfers to biotin in an ATP-dependent reaction. Subsequently, the biotinyl group serves as a temporary carrier of CO_2_, transferring it to acetyl-CoA in the second step to yield malonyl-CoA [28].

FAs are synthesized from acetyl-CoA by a cytosolic complex of six enzyme activities plus acyl carrier protein (ACP), which proceeds in a repeating four-step reaction sequence that includes condensation, reduction of the carbonyl group, dehydration, and reduction of the double bond. For example, the synthesis of palmitate (C16:0) requires seven cycles of condensation and reduction (Equation (1)). The overall stoichiometry of glucose conversion to stearic acid and TAG synthesis is approximated in Equations (2) and (3) below. Therefore, if glucose is not used for the synthesis of any other product, the lipid yield is approximately 32 g per 100 g of glucose [14].

8 acetyl-CoA + 7 ATP + 14 NADPH + 14 H^+^ → palmitate + 8 CoA + 7 ADP + 7 Pi + 14 NADP^+^ + 6 H_2_O (1)

4.5 Glucose + CoA + 9 NAD^+^ + 7 NADPH + 17 ATP → C18-fatty acyl-CoA + 9 CO_2_ + 9 NADH + 7 NADP^+^ + 17 ADP + 17 P_i_ (2)

15 Glucose → Triacylglycerol (with stearic acid) + 36 CO_2_ (3)

All reactions in the synthetic process are catalyzed by a multienzyme complex, FA synthase (FAS). In some organisms, FAS consists of multifunctional polypeptides, which function as carriers of the fatty acyl intermediates. Although the details of the enzyme structure differ between prokaryotes (*E. coli*) and eukaryotes (*S. cerevisiae*), the four-step process of FA synthesis is the same [28]. Synthesis of FA requires acetyl-CoA and the input of chemical energy in two forms: ATP to power the joining of CO_2_ to acetyl-CoA to make malonyl-CoA and NADPH to reduce the double bonds. The level of malonyl-CoA formation could be an important factor in regulating the biosynthesis of FAs [28].

Most FAs synthesized or ingested by yeast have one of two fates for incorporation: incorporation into triacylglycerols (TAGs) for the storage of metabolic energy or incorporation into the phospholipid components of membranes. TAGs have the highest energy content of all stored nutrients—more than 38 kJ/g [28]. In yeasts, TAG synthesis occurs via the Kennedy pathway [31] in two phases: one is diacylglycerol (DAG) formation; another is the acylation reaction that converts DAG to TAG. There are two different pathways to accomplish *de novo* synthesis of DAG in yeast, namely the glycerol-3-phosphate (G3P) pathway and the dihydroxyacetone phosphate (DHAP) pathway (Figure 1). In the first step of TAG assembly, lysophosphatidic acid (LPA) can be acylated from G3P in the *sn-1* position by G3P acyltransferase (*SCT1*) directly or formed via DHAP by the combination of G3P acyltransferase, DHAP acyltransferase, and 1-acyl DHAP reductase. Then, LPA is acylated to phosphatidic acid (PA) in the *sn-2* position by LPA acyltransferase (*SLC1*), followed by dephosphorylation of PA by phosphatidate phosphatase (PAP) to yield DAG. In the last step, the third acyl group can be added at the *sn-3* position either through the acetyl-CoA dependent pathway (DAG acyltransferase (DGAT), DGA1 with acyl-CoA as an acyl donor) or through the acyl-CoA-independent pathway (phospholipid DAG acyltransferase (PDAT), *LRO1* with glycerophospholipids as an acyl donor) [32,33]. Furthermore, TAGs can also be produced from FAs on DAGs in a phospholipase A_2_-dependent deacylation–reacylation mechanism [34].

In contrast to FA accumulation, FA degradation occurs via the β-oxidation pathway in peroxisomes in yeast cells. FA degradation can happen during the exponential phase of cell growth to provide energy for membrane lipid synthesis, cellular growth, and division, during the stationary phase to overcome the lack of environment nutrients, and during the phase where cells exit starvation conditions [35].

Surplus neutral lipids, such as TAG, are sequestered into LDs. In the 19th century, LDs were first identified by light microscopy as cellular organelles and have been referred to as lipid bodies, fat bodies, oil bodies, spherosomes, or adiposomes. LDs have been ignored for a long time as inert lipid globules with little functional relevance before being recognized as metabolically highly active organelles. LDs form the main lipid storage organelles for neutral lipids in eukaryotic cells, such as *S. cerevisiae* and even some prokaryote cells, such as *Mycobacteria, Rodococcus, Streptomyces,* and *Nocardia* [29,36]. LDs are primarily composed of TAG and sterol ester (SE) (roughly 50% each by weight) with a small amount of phospholipids in the wild-type yeast [37], and its formation and utilization is not essential to *S. cerevisiae* [38]. These LDs are thought to be formed in the endoplasmic reticulum (ER) and surrounded by a phospholipid monolayer with a highly hydrophobic core. LDs vary dramatically in size but have an approximate diameter of 0.1 µm in yeast [29,30]. It is not known precisely how the bi-layered membrane gives rise to LDs enclosed by monolayers, but several models of LD biogenesis have been proposed. For example, (1) an ER budding model (also the most widely cited model) where LDs grow from the endoplasmic reticulum (ER) bilayer and remain connected or bud off; (2) bicelle formation, in which an entire lipid lens is excised from the ER [39]; (3) vesicular budding in which a bilayer vesicle forms, followed by filling the bilayer intramembranous space with neutral lipids [30]; and (4) an “eggcup” model in which an LD grows within a concave depression of the ER through transport of neutral lipids form the ER [29,40].

## 2. Modular Metabolic Engineering Strategies for Standard and High-Value Lipid Production

In general, lipid production is optimized by blocking competing pathways that consume lipids, FFAs, and other intermediates, strengthening the lipid synthesis pathway, and keeping a balance between the synthesis of precursors, intermediates, and cofactors [4]. To increase lipid production in yeast, there are several approaches using genetic engineering, such as (1) increasing FA biosynthesis via overexpression of acyl-CoA carboxylase (ACC), Acyl-CoA synthase (ACS), fatty acid synthase (FAS) genes; improving energy supply, such as NADPH and ATP via pathway engineering; deleting genes for FA consumption pathways, such as β-oxidation pathway; (2) increasing TAG synthesis via overexpressing acyl-transferases including lysophophatidate acyl-transferase (LPAT), acyl-CoA: diacylglycerol acyl-transferase (DGAT) and other related pathway enzymes; (3) enhancing LD biogenesis and stability through overexpression of LD-associated genes/proteins and deletion of LD consumption-related proteins/genes or precursors; and (4) the combination of the first three modular strategies to improve lipid production [41,42,43].

### 2.1. Increase Fatty Acid Biosynthesis Module

#### 2.1.1. Acetyl-CoA Synthase

Acetyl-CoA synthase (ACS) catalyzes the formation of acetyl-CoA from acetate and CoA, which is the starting point of fatty acid biosynthesis. Chen et al. overexpressed both *ACS1* and *ACS2* in *S. cerevisiae* to increase the intracellular acyl-CoA levels about 2 to 5 times [44]. ACS variant (*SEACS^L641P^*) was reported to be critical for preventing the acetylation of ACS by protein acetyltransferase and maintaining the ACS enzyme in its active state in *S. enterica* [45]. Also, the activity of *SEACS^L641P^* was demonstrated in *S. cerevisiae* by successful redirection flux from acetaldehyde to acetyl-CoA in the cytosol [46]. Then, this ACS variant (*SEACS^L641P^*) has been popular used to produce either lipid [47] or lipid-derived products [48,49] that need acetyl-CoA as precursor.

#### 2.1.2. Acetyl-CoA Carboxylase

The enzyme acetyl-CoA carboxylase (ACC) catalyzes acetyl-CoA from malonyl-CoA, which is an important rate-limiting step in FA biosynthesis. The Keasling group showed that plasmid-based overexpression of endogenous *ACC1* increased lipid content by 58% in *S. cerevisiae* from 4.3% to 6.8% by DCW [26]. However, no significant increase in total FA content was observed by plasmid-based overexpression of wild-type *ACC1* in *S. cerevisiae* by the Nielsen group [50]. The possible reason for the limited effects of *ACC1* overexpression may be the inherently low enzyme activity. In addition, ACC activity can be inactivated by *Snf1*- or other mediated phosphorylation in yeast, so a malonyl-CoA sensor could be used to screen phosphorylation sites to improve malonyl-CoA-derived products [51]. Furthermore, the Nielsen group engineered the mutant version *ACC1^ser659ala,ser1157ala^*, showed 3-fold improved ACCase activity, and increased the total lipid content by 65% [50]. Interestingly, the Da Silva group engineered another mutant version *Acc1^ser1157ala^* and demonstrated 3-fold improvement in both polyketide and fatty acid production [52].

#### 2.1.3. Fatty Acid Synthase

Another important enzyme involved in FA biosynthesis is the FA synthase (FAS) complex. It receives acetyl and malonyl groups for the subsequent elongation and final formation of acyl-CoAs. In *S. cerevisiae*, the FAS is composed of two non-identical subunits with multiple functions encoded by the genes *FAS1* and *FAS2*, respectively. Plasmid-based overexpression of both *FAS1* and *FAS2* in yeast led to a 30% increase in lipid content (from 42.7 mg/L to 70.6 mg/L) [26]. As FAS is a large molecular weight, multi-enzyme complex with subunits closely related to each other, and multipoint controls differing with species, these together are a challenge for employing this complex in lipid engineering strategies.

#### 2.1.4. β-Oxidation Pathway

Blocking FA consumption via the β-oxidation pathway is a logical strategy to provide more FAs. In *S. cerevisiae*, the β-oxidation pathway was engineered by deleting gene *POX1* and overexpressing *POX2* gene from *Yarrowia lipolytica* to generate strain △*POX1* [*POX2*+], which increased the total FAs by 29.5%, 2.26-fold higher intracellular FA than the wild type [53]. Similarly, inhibition of β-oxidation (*POX1* deletion) led to a 4-fold increase in FFA content in *S. cerevisiae* [54]. However, as the β-oxidation pathway provides the acetyl-CoA, NADH, etc. precursors and energy, a potential disadvantage of deleting the β-oxidation pathway is the result of slow cell growth, which has been reported for deletion of *POX1* in *S. cerevisiae* [53].

#### 2.1.5. NADPH Supply

In addition to the precursor acetyl-CoA, chemical energy in the form of ATP and NADPH is also important because fatty acid biosynthesis is a highly energy-consuming process. The pentose phosphate pathway is a main source of NADPH generation for FA biosynthesis in yeast, which is constrained by the supply of NADPH [55,56]. This suggests FA biosynthesis could be improved by increasing NADPH supply. Engineering the cytosolic redox metabolism to increase the NADPH supply improved the lipid content to 90 g/L in *Y. lipolytica* [57]. In *S. cerevisiae*, NADPH limitation could be overcome by the expression of isocitrate dehydrogenase, which was demonstrated by improved α-ketoglutarate production [58]. Furthermore, fine-tuning of NADPH and ATP supply, together with other metabolic engineering strategies to replace alcoholic fermentation with lipogenesis in *S. cerevisiae*, achieved the highest 33.4 g/L extracellular free fatty acids [59]. This approach was not limited to yeast; two examples where NADPH supply was enhanced in microalgae improved lipid production [60,61].

### 2.2. Enhance the Lipid Accumulation Module

Acyltransferases are the key enzymes that catalyze the addition of fatty acids to the glycerol backbone of triacylglycerol or phospholipids. There are important acyltransferases in lipid accumulation, including glycerol-3-phosphate acyltransferase (GPAT), lysophophatidate acyltransferase (LPAT), diacylglycerol acyltransferase (DGAT), and phospholipid: diacylglycerol acyltransferase (PDAT).

#### 2.2.1. Glycerol-3-Phosphate Acyltransferase

GPAT catalyzes the first step in TAG synthesis, the conversion of glycerol 3-phosphate and acyl-CoA to lysophophatidate (LPA). The overexpression of GPAT from cocoa in *S. cerevisiae* effectively increased the total lipid content 1.3-fold with more cocoa butter, such as TAG [62]. Expression of phosphorylation-deficient GPAT Gpt2 increases the cellular TAG level, and phosphorylation of Gpt2 at a conserved motif plays a critical role in coordinating the synthesis and degradation of TAGs [63].

#### 2.2.2. Lysophosphatidate Acyltransferase

LPAT assists lysophosphatidate (LPA) acylate to form phosphatidate (PA), which is an important precursor for TAG synthesis (Figure 1). The overexpression of LPAT from cocoa, cotton, and sunflower effectively increased the TAG content and total lipid content in *S. cerevisiaei* [62,64,65]. In addition, the expression of yeast LPAT (*SLC1-1*) was demonstrated to increase oil content by 6-fold on a dry seed weight basis in *A. thaliana* and *Brassica napus* [66].

#### 2.2.3. Diacylglycerol Acyltransferase

DGAT catalyzes the last step of TAG synthesis from DAG and fatty acyl-CoA, which has been shown to be a very effective lipid accumulation enzyme. When an extra copy of yeast *DGA1* was expressed in yeast under the control of a strong constitutive promoter, lipid content was increased 1.5-fold [26]. Heterologous expression of DGATs from plants including rice, cocoa, *A. thaliana*, *B. napus*, *Linum usitatissimum*, and the fungi *Mortierella ramanniana* in *S. cerevisiae* also increased TAG or lipid content [67,68,69,70,71,72].

#### 2.2.4. Other Potential Lipid Accumulation Enzymes

Phospholipid: diacylglycerol acyltransferase (PDAT) catalyzes the acyl-CoA independent formation of TAG using phospholipid and DAG as substrates [73]. PDAT was also reported to have overlapping functions with DGAT for TAG synthesis in *Arabidopsis* [74]. PDAT overexpression increased 29–47% of total fatty acids [73] and rescued oleic acid sensitivity and TAG accumulation in yeast [75], which was also applied to a recent study expressing PDAT from *Sapium sebiferum* (L.) *Roxb* [76].

Additionally, the enzyme PC: DAG phosphocholine transferase (also known as Reduced Oleate Desaturation, ROD1) transfers a phosphocholine headgroup of PC to the *sn*-3-position of a DAG molecule. The ROD1 from *Arabidopsis* was identified by its action in significantly increasing the content of polyunsaturated fatty acid (PUFA) in oil but not overall oil content [77], and a similar trend was found after heterologous expression of *AtROD1* in *S. cerevisiae* [67].

Although expressing acyltransferases is generally effective in increasing the lipid or TAG content in the yeast cell because acyltransferases catalyze the addition of fatty acids to the glycerol backbone of triacylglycerol or phospholipids, there is a limitation in the improvement of the lipid titer due to the limited carbon and precursor flux toward the lipid accumulation module. This limitation could be overcome by combining acyltransferase expression with other lipid production modules.

### 2.3. Improve the Lipid Sequestration Module

#### 2.3.1. Increase Neutral Lipid Supply

As described earlier, TAG is synthesized and then sequestrated in the form of LDs, which contain a hydrophobic core composed of TAG and sterol esters (SE), surrounded by a monolayer of phospholipids. Five enzymes are responsible for the mobilization of stored lipids in yeast: TAG lipases, including Tgl3p, Tgl4p, and Tgl5p, and SE hydrolases, including Yeh1p and Tgl1p. Tgl3p and Tgl4p are the major TAG lipases in yeast; deletion of these genes leads to markedly increased LD size and number, whereas Tgl5p only marginally contributes to TAG hydrolysis under standard growth conditions [78]. Reduction in TAG hydrolysis via the deletion of *tgl3* or *slc1* (*slc1* encodes a 1-acylglycerol-3-phosphate acyltransferase involved in phosphatidic acid biosynthesis) increased LD production [79], which was consistent with previous research findings that the deletion of *tgl3* or *slc1* could increase TAG levels in cells [80,81]. Whereas deletions of sterol synthesis genes (*HMG2*, *ERG4,* and *ERG5*), TAG synthesis gene (*DGA1*), and SE synthesis gene (*ARE2*) all showed reduction in LD contents [79].

#### 2.3.2. Lipid Droplet Stabilization

On the surface of LDs, the phospholipid monolayer membrane contains a small content of embedded proteins, which assist in the formation of LDs and are called lipid droplet-associated proteins (LDAPs) [78]. Several proteins of yeast LD have putative assignments for involvement in phosphatidic acid biosynthesis, FA activation, TAG, and SE metabolism, and sterol synthesis. In contrast to other cellular organelles, the monolayer membrane of LDs is distinctive [82]. The prominent LDAPs in mammalian cells are perilipin, adipophilin, and adipose TG lipase (ATGL), while oleosins are the most prominent proteins of plant oil droplets; these help cover the surface of droplets and prevent them from coalescence [83,84]. Some LDAPs have been identified in the formation and turnover of LDs, such as SNAP23, LDAP1, LDAP2, and At3g0550 in avocado (*Persea americana*) mesocarp and *A. thaliana* [85], and some proteins have been associated with lipid accumulation in oil-rich fruit tissues [86]. Since LDAPs are important for covering the droplet in plants, they may also be important in the yeast LD formation mechanism. Interestingly, yeast LDs do not contain proteins related to the perilipin family of proteins in mammals or oleosins in plants, raising questions as to the formation and stabilization of LDs in yeast without associated proteins [78].

There are several yeast LDAP-related proteins or factors, including Fld1p (encodes seipin), fat-inducing transcript (FIT), Pah1p (yeast lipin orthologue), phosphatase, phospholipid, phosphatidic acid, etc., which have shown an effect on the formation, morphology, size, or number of LDs [79,85,87,88]. *Fld1*, the functional orthologue of the human *BSCL2* gene encoding seipin, is important to LD morphology, subcellular distribution, and inheritance, and deletion of *Fld1* in *S. cerevisiae* leads to impaired dynamics of yeast LDs, defective lipolysis, and causes abnormal LDs [29], which might be due to aberrant ER structures [89]. *Fld1* was identified among ten yeast mutants producing “supersized” LDs that were up to 50 times the volume of those in WT cells [88].

Another two proteins, fat-inducing transcript (FIT) and yeast lipin orthologue of Pah1p, have been reported to help LD formation. FIT are ER proteins that bind TAG and have been implicated in LD assembly. FIT2 overexpression in yeast achieved more LDs, while the deletion of the genes resulted in fewer LDs and TAG, but there is no evidence that FIT proteins affect DGAT activity [90]. The yeast lipin orthologue of Pah1p was reported to control the formation of cytosolic LDs. Disruption of *Pah1* resulted in a 63% decrease in LDs number, so it was concluded that DAG generated by Pah1p is important for LD biogenesis [91].

### 2.4. Fatty Acid Modification Module towards High-Value Lipid Production

Cyclopropane fatty acids (CFAs) are modified fatty acids possessing a unique strained ring structure that conveys oxidative stability and lubricity to lipids where they are incorporated. Such lipids have applications as high-value cosmetics and in specialist lubrication. Yeast *S. cerevisiae* has been popularly employed by the plant research team to test the effectiveness of plant- or bacterial-sourced cyclopropane fatty acid synthetases (CFAS) by the plant research team [92,93,94,95]. CFAS sourced from *Escherichia coli* (*Ec. CFAS*) was found to be an effective enzyme for CFA synthesis and has been used in several yeasts, including *S. cerevisiae* and *Yarrowia lipolytica*. We successfully transferred the lipid pathway engineering strategies from standard lipids to high-value CFA synthesis. We expressed the *E. coli CFA* gene in *S. cerevisiae* that had been engineered for higher fatty acid (FA) biosynthesis, lipid production, and sequestration. *TGL3*, encoding triglyceride lipase 3, the main enzyme responsible for hydrolyzing CFA from TAG, was knocked out to block CFA loss from the lipid droplet. The highest CFA yield was 12 mg/g dry cell weight (DCW), which was 4-fold above the strain expressing *E. coli CFA* gene only and up to 68.3 mg/L in a two-stage bioprocess. We also compared the distribution of CFA between neutral lipids and phospholipid in yeast *S. cerevisiae*, which is rarely reported by others [47,96]. Moreover, the expression of *Ec. CFAS* in heavy engineered *Yarrowia lipolytica* achieved >32% CFA of total lipids, and titer reached 3.0 g/L in the bioreactor [97].

Ricinoleic acid (RA) is an unsaturated omega-9 fatty acid with a hydroxyl group in the ∆12 position, and a major fatty acid in triglyceride form of castor oil seed [98]. Similar to CFA, RAs have many industrial applications, such as in lubricants, nylon, dyes, ink, etc., and its production has been conducted on a commercial scale for many decades. Issues stemming from oilseed castor cultivation and harvest, such as dermatitis among field workers and the presence of deadly ricin toxin, make it necessary to develop microbial sources to produce RAs [99]. In the fission yeast *Schizosaccharomyces pombe*, *CpFAH12* was expressed to bring RA production to 137.4 μg/mL [99]. Moreover, the Uemura group engineered the *Schizosaccharomyces pombe* to secrete RA and reduce its toxicity to cells [100,101]. The Nicaud group engineered *Yarrowia lipolytica* by co-expression of *CpFAH12* and *PDAT* to accumulate RA to 43% of total lipids, and over 60 mg/g DCW [102]. RA accumulation attempts were also tested in microalgae and plants by expressing castor LPAT or *CpFAH* [103,104].

Gamma linoleic acid (GLA) is a high-value polyunsaturated fatty acid containing 18-carbon chain and three cis double bonds in the ∆^6,9,12^ positions. GLA is regarded as an essential fatty acid in the diet because mammals lack the ability to synthesize it, and it is important to regulate normal physiological functions [105]. Co-expression of Δ12- and Δ6-desaturases from *Mortierella alpina* in *S. cerevisiae* achieved 8% GLA of total fatty acids, and GLA was accumulated predominantly in the phospholipid fraction [105].

Other important high-value fatty acid examples are dietary omega-3 acids, such as eicosapentaenoic acid (EPA) and docosahexaenoic acid (DHA), which belong to the long chain polyunsaturated fatty acids (carbon chain lengths between 20–24). EPA and DHA are obtained mainly from fish oil, and fish stock depletion makes it necessary to develop the EPA and DHA producing sustainable microbial cell factories toward the increasing market [106]. Compared with the oleaginous yeast, such as *Yarrowia lipolytica* and *Lipomyces starkeyi*, *S. cerevisiae* is not a promising host to achieve a high EPA or DHA titer. For example, DuPont has engineered the yeast *Yarrowia lipolytica* to achieve EPA titer at 25% DCW and at more than 50% lipids, plus two commercial products, New Harvest™ EPA oil and Verlasso^®^ salmon [107]. However, *S. cerevisiae* has always been an important host to understand or evaluate the key enzymes or steps in the EPA and DHA synthesis pathways; for example, the desaturases and elongases are very important for the very long chain polyunsaturated FA biosynthesis reconstituted in yeast [108].

### 2.5. Combination of Lipid Pathway Engineering with Other Emerging Engineering Strategies

Well-targeted single gene or pathway modifications in yeast normally lead to increased lipid content, although this improvement is limited; for more considerable improvement in lipid production, it is necessary to combine strategies, such as the “push-pull-block” strategy: (1) pushing the central carbon flux toward fatty acid biosynthesis, such as enhancing the biosynthesis of precursors, such as acetyl-CoA, malonyl-CoA, and fatty acyl-CoA pool; (2) pulling the free fatty acid towards stored lipids (triacylglycerol) by expressing diacylglycerol acyltransferase, lipid droplet associated protein, etc.; and (3) blocking the fatty acid and lipids degradation pathway, such as by deleting the beta-oxidation pathway or TAG lipase [109,110]. Several multi-pathway attempts have been undertaken by researchers to enhance (extracellular and intracellular) lipid production in *S. cerevisiae,* and here, only recent examples with the maximum lipid yield are summarized. For the extracellular lipids, the Da Silva group achieved 2.2 g/L extracellular FFAs through disrupted neutral lipid recycle in *S. cerevisiae,* including disruption of β-oxidation and FA accumulation △*FAA2*, *PXA1*, *POX1*, *FAA1*, *FAA4, FAT1*, and co-expression of *DGA1* and *TGL3* [111]. In 2016, the Nielsen group reached 10.4 g/L extracellular FFAs by enhancing the acetyl-CoA pathway, malonyl-CoA pathway and reverse pathway and disrupting FA accumulation [112]. Furthermore, a recent breakthrough from the Nielsen group through reprogramming yeast metabolism from alcoholic fermentation to lipogenesis constructed a synthetic oil yeast that could produce up to 33.4 g/L FFAs [59]. In terms of intracellular accumulation, notably, the Nielsen group reported the highest TAG content of 254 mg TAG/g DCW in *S. cerevisiae* as per the gene combination shown in Table 2 and reached 27.4% of the maximum theoretical yield [110]. These breakthroughs have employed the latest CRISPR genome editing tools to prepare the engineering strains and have used Adaptive Laboratory Evolution (ALE) strategies to further improve the lipid content and titer. A summary of the achievements in lipid production in yeast through combinations of engineering approaches is summarized in Table 2.

While the metabolic lipid pathway engineering of an organism is the primary step toward creating a lipid production biofactory, apart from lipid yield and titer, volumetric productivity is a key performance indicator for successful lipid production. For intracellular production of lipids, volumetric productivity is limited by biomass yield due to the inherent trade-off between the production of biomass and lipids, and biomass growth is easily impaired during lipid pathway modification. To improve lipid productivity, bioprocess strategies are commonly employed, especially the two-stage strategy, which uncouples lipid production and biomass growth. This can be achieved through the inducible expression of genes coding for enhanced lipid productivity being turned on after the biomass growth stage has been achieved [47]. However, the productivity in the two-stage bioprocess is not automatically better than a single-stage bioprocess, but it is dependent on substrate uptake rate and utilization rate [113]. Other bioprocess strategies that are employed to improve yeast biomass and lipid productivity include the use of altered C:N ratio in a second stage of the bioprocess [114]. In addition, protein engineering, such as the engineered ScFAS, bacterial type I FAS, plus directed evolution of membrane transporter Tpo1, strain adaptive laboratory evolution, and combined with an optimized cultivation process achieved a >1 g/L extracellular medium chain fatty acids (MCFAs)—a more than 250-fold improvement over the original strain [115]. With the rapid development of synthetic biology tools, such as modular cloning systems, CRISPR genome editing tools, and machine learning guided artificial intelligence design of microbial factories, especially the global alliance of biofoundries [116], these could provide more possibilities that promote the integration and automation of the design, build, test, and learn (DBTL) modules of microbial cell factories for lipid production.

**Table 2:** Metabolic engineering strategies of yeast *S. cerevisiae* for improved lipid production.

Gene/Enzyme Modification	Remarks/Achievements	Refs
**Improved fatty acid biosynthesis module**		
↑ *ACC1*	↑ 58%, 6.8% lipids	[26]
(1)↑ *ACC1* (2)↑ *ACC1*^ser659ala,ser1157ala^	(1) Not significant (2) ↑ 65% FAs	[50]
*ACC1* ^ser1157ala^	↑ 3-fold FAs	[52]
*ACC1* ^ser659ala,ser1157ala^	Not significant	[47]
↑ *FAS1*, *FAS2*	↑ 30% lipid content, 70.6 mg/L (5.6% CDW)	[26]
↑ *ACS1*, *ACS2*	↑ 2–5 × acetyl-CoA level	[44,49]
↑ *SeACS*^L641P^	↑ α-santalene	[49]
↑ *ACS1*	↑ acyl-CoA level ↑ 8–23% amorphadiene	[46]
↑ *SeACS*^L641P^, *ADH2*, *ALD6*, *WS2*	↑ 3 × FAEE, 408 ± 270 ug/g CDW	[117]
(1) △ *POX1*, ↑ *POX2;* (2) △ *POX1*, ↑ *POX2*, ↑ crot	(1) ↑ 29.5% total FAs, 2.26 × intracellular MCFAs, 3.29 × extracellular MCFAs; (2) ↑ 15.6% total FAs, 1.87 × intracellular MCFAs, 3.34 × extracellular MCFAs	[53]
△ *POX1*	↑ 4 × FFAs	[54]
△β-oxidation, △*ACSs*, △*ADH1*, ↑ thioesterases, ↑ *ACC1*, ↑ acetyl-CoA	↑ 2 ×, 140 mg/L FAs	[27]
(1) △β-oxidation, △*FAA2*, *PXA1, POX1*; (2) △*ACSs*, *FAA1*, *FAA4*, *FAT1*; (3) Combine (1) & (2)	(1)↑ intracellular FAs up to 55%; (2)↑ extracellular FFAs to 490 mg/L; (3)↑ 1.3 g/L extracellular FFAs	[111]
△*FAA1*△*FAA4*, ↑ acyl-CoA thioesterase *ACOT5* (Acot5s)	↑ 6.43 × FFAs, 500 μg/mL, ↑ UFA ratio (42% > 0 in WT), ACOT5 helps restore cell growth	[118]
(1) △*ARE1*△ *DGA1* △*ARE2*△*LRO1*; (2) △*POX1*; (3) Combine (1) & (2)	(1) 3 × FFA; (2) 4 × FF; (3) 5 × FFA	[54]
↑ *ACC1*, ↑ *FAS1*, ↑ *FAS2*	>17% DCW lipids, ↑ 4× than WT	[26]
**Improved lipid accumulation and sequestration modules**		
** ↑ ** ***At-Gh13LPAAT5***	↑ 25–31% in palmitic acid and oleic acid; ↑ 16–29% TAG	[64]
** ↑ ** ***DGAT***	↑ 3–9 × TAG, (25- 80 nmol TAG/mg DCW)	[16]
↑ Dga1p (YOR245c)	↑ 70–90 × DGAT activity in LDs; ↑ 2–3 × in ER.	[69]
↑ *DGAT1*, ↑ N-terminal tag	↑ 53% × TAG, 28% × total FAs, 453 mg FAs/L	[70]
↑ *PDAT*	↑ TAG, 2 × (log phase), 40% × (stationary phase), identified PDAT gene YNR008w	[73]
* ↑ * *LuPDCT1, LuPDCT2,* * ↑ * *FAD2, FAD3*	↑ PUFAs (linoleic acid (18:2 cisΔ9,12), α-linolenic acid (18:3 cisΔ9, 12, 15)) levels in phosphatidylcholine (PC), DAG, and TAG	[71]
△*SLC1* (YDL052C); △*TGL3* (YMR313C)	↑ LD content	[79]
△*HMG2* (YLR450W); △*DGA1*(YOR245C); △*ERG4* (YGL012W); △*ERG5* (YMR015C); △*ARE* (YNR019W); △*SIT4* (YDL047W); △*REG1* (YDR028C); △*SAP190* (YKR028W)	↓ LD content	[79]
△*PAH1*	↓ 63% × LDs number, total lipids stable	[91]
↑ WS2, ACB1, GAPN	↑ 7.7×, 48 mg/L FAEE	[119]
**Combination of lipid production modules plus other engineering solutions**		
↑ *ACC1*, *FAS1*, *FAS2*, terminal “converting enzymes”	400 mg/L FFA, 100 mg/L fatty alcohols, 5 mg/L FAEE	[26]
(1) ↑ *ACL*, △*IDH1*; (2) ↑ *ACL*, △*IDH2*; (3) ↑ *ACL*, △*IDH1*, *2*	(1) ↑ 80% C16:1, ↑60% C18:1; (2) ↑ 60% C16:1, ↑45% C18:1; (3) ↑ 92% C16:1, ↑77% C18:1	[120]
(1) ↑ Reversed β–oxidation pathway, *SeACS^L641P^*, △*ADH1, 4*, △*GPD1,2*; (2) ↑ Reversed β-oxidation pathway, *EEB1* or *ETH1*	(1) ↑ medium-chain FAEEs (0.011 g/L FFA, C16, C18); (2) ↑ FAEE (C4–C10, 0.75 g/L)	[121]
(1)↑ *WS2*, *ADH2*, *ALD6, SeACS^L641P^*; (2)↑ *WS2*, xpkA, ack, pta	(1) ↑ 3 × FAEE (408 ± 270 μg/g, DCW) (2) ↑ 1.7-fold FAEE (5100 ± 509 μg/g, DCW).	[117]
↑ *WS2*, △ *FAA2*, △*ACB1*, △*PXA2*	↑ 17×, 25 mg/L FAEE	[122]
△β-oxidation, △*FAA2*, △*PXA1* & △*POX1*, △*ACSs*, △*FAA1*, 4 & △*FAT1*, ↑*DGA1* & ↑*TGL3*	2.2 g/L extracellular FFAs	[111]
(1) △*SNF2*; (2) ↑*LEU2*, △*SNF2*; (3) ↑*DGA1*, △*SNF2*; (4) ↑*LRO1*, △*SNF2*; (5) ↑*FAA3*, ↑*DGA1*,△*SNF2*	(1) ↑ lipid; (2)↑ growth and lipid accumulation; (3)↑ lipids; (4)↓ lipids; (5)↑lipids, 30% lipids content, mainly TAG (add exogenous FAs).	[123]
↑ *ACC1^ser659ala,ser1157ala^*, ↑*PAH1*, ↑*DGA1*, △ *GUT2*, △*ARE1*, △*PXA1*, △*POX1*, △*FAA2*, △*TGL3*, 4, 5	254 mg TAG/g DCW, 27.4% of the maxi theoretical yield	[110]
Engineering ScFAS, bacterial type I FAS, directed evolution of membrane transporter Tpo1, strain adaptive laboratory evolution	↑ 250-fold, >1 g/L Medium-chain fatty acids (C6–C12)	[115]
↑ *RtME*, ↑*MDH3*, ↑*CTP1*, ↑*MmACL*, ↑*RtFAS*, ↑*ACC1*, ↑*tesA*, △*POX1*, △*FAA1, 4*	10.4 g/L extracellular FFA	[112]
↑ Cytosolic acetyl-CoA, ↑ NADPH supply, ↑ FA biosynthesis, △ethanol pathway, mutate pyruvate kinase and direction evolution: ↑MPC, *RtCIT1, ME, PYC1, YHM2, MDH3, RtFAS, ACC1, AnACL, MmACL, IDP2,* *↓PGI1, IDH2,* *↑* *ZWF1, GND1, TKL1, TAL1*, etc.	33.4 g/L extracellular FFA, the highest titer reported to date in *S. cerevisiae*	[59]

‘↑’, overexpression or heterologous expression, increase; ‘↓’: downregulation or reduce; ‘△’: deletion or knockout, ‘×’, times by folds.

## 3. Cellular Physiological Responses to Lipid Pathway Engineering

Lipid pathway engineering is not a simple calculation or combination of different modules of lipid production, but it will also take care of cellular physiological responses to avoid the metabolic burden, especially the linkages between engineering strategies, lipid production, and lipotoxicity. Excess or limited precursors in each module, such as acetyl-CoA, malonyl-CoA and FFA, could be toxic to the cell and cause the cell sick. It is important to consider the central carbon metabolism, lipid metabolism, and lipotoxicity together to raise feasible engineering strategies to address the cellular physiological responses. Since lipids can be used for membrane synthesis and energy storage in yeast cells, lipid pathway engineering inevitably will have some impact on cell growth and replication, intracellular biochemical processes, and energy balances. These effects can be manifested in cellular physiological differences between wild-type and engineered cells, including cell growth, cell membrane integrity, reactive oxygen species, and mitochondria membrane potential, and heterogeneity.

### 3.1. Cell Growth

Cell growth means cell reproduction or growth in cell populations, which is often coupled to cell division and contains three phases: first growth phase, synthesis phase, and second growth phase [124]. Cell growth is routinely measured by optical density at 600 nm in the liquid cell culture or colony-forming units on an agar plate [125]. Additionally, another two important parameters, including the specific growth rate (µ) and duplication (double) time, are usually used to evaluate the dynamic behavior of cell growth. FA biosynthesis is one of the prerequisites for yeast cell growth and cell division due to its importance in membrane synthesis. Imbalance in the synthesis or turnover of lipids affects yeast growth and viability [126]. Many biologically critical functional proteins that control Na^+^, K^+^, and pH homeostasis are housed by lipid and membrane rafts, and effects on these could influence cell growth [127]. In addition, supplying more malonyl-CoA by overexpressing *ACC1*^ser659ala,ser1157ala^ to enhance FA biosynthesis severely reduced to two-thirds of yeast cell growth [50]. Lipotoxicity, a metabolic syndrome, is caused by the accumulation of FFA, leading to cellular dysfunction and death. Thus, the connection of lipids to cell growth and cell death is more complicated than solely the lipotoxic effects of excess free fatty acids. In addition, saturated FA did not affect cell growth, while shorter chain FA or highly unsaturated FA impaired cell growth and caused ROS accumulation and activation of the unfolded protein response [128]. However, neither storage lipids nor lipid bodies are essential for yeast cell growth because no apparent growth defects were found in the yeast strain lacking all four genes, including *ARE1*, *ARE2*, *DGA1,* and *LRO1* [38]. Moreover, TAG accumulation has been reported to protect against fatty acid-induced lipotoxicity [129].

### 3.2. Cell Membrane Integrity

Cell membrane integrity means the cell membrane can protect intracellular organelles, such as the nucleus, mitochondria, and ribosomes, and regulate the entry of substances, such as nutrition and toxins passing through the cell. A break in the cell membrane immediately compromises its essential role in protecting the cell and may cause cell death [130]. In addition, cell membrane integrity indicates the ‘cell health’ [131], is closely related to cell growth, and is an important response to lipid pathway engineering. Compromised cell membranes cannot generate an electrochemical gradient, or membrane potential, which is generated by the regular passive and active transport across the intact cell membrane [132] and may not act as a barrier against the loss of essential cellular electrolytes. The cell membrane lipid composition affects its integrity to impact the ethanol tolerance of yeast cells [133]. In addition, sphingolipids and sterols in the yeast cell membrane coordinately regulate cell membrane integrity [134]. The membranes of yeast may also be affected by metabolites secreted into the cell culture medium, such as acetic acid and ethanol [135]. Furthermore, the fatty acid unsaturation degree and chain length affect the cell membrane fluidity of *S. cerevisiae* and hence the organism’s resistance to metabolites in the medium [136,137]. Measurement of cell membrane integrity is routinely detected by flow cytometry with propidium iodide (PI) staining, as the dye only penetrates permeable membranes where it binds to DNA and fluoresces [138,139].

### 3.3. Reactive Oxygen Species

Due to the reactive nature of molecular oxygen, reactive oxygen species (ROS) include either oxidants, such as peroxide and superoxide, or reductants, such as hydroxyl radicals [140]. Most endogenous ROS production in yeast cells is generated by the mitochondria [140], which originates from the leakage of electrons from the respiratory transport chain as an ordinary consequence of aerobic respiration [141]. If the ROS levels exceed cellular antioxidant defenses, such as specific enzymes and metabolites like glutathione, oxidative damage will be applied to proteins, lipids, and DNA, and these may impair cell membrane integrity [142]. Mitochondria can generate antioxidant enzymes, such as glutathione-s-transferase (GSTs) and manganese-dependent superoxide dismutase (Mn-SOD), to reduce or eliminate the presence of ROS [140]. ROS generation in the cell may be detected by cell-permeant fluorescent dye CM-H_2_DCFDA, which is first cleaved by an intracellular esterase, although the technique has not been widely implemented for ROS detection in yeast [143].

While a mechanistic link between enhanced lipid synthesis and oxidative stress in yeast has not yet been identified, there are studies drawn from yeast and mammalian systems to support the existence of such a relationship. The FFA metabolite ceramide can activate NADPH oxidase and disrupt mitochondrial respiration, or FFAs can cause endoplasmic reticulum stress and indirectly lead to oxidative stress [142]. Drawing on human and experimental animal research, oxidative stress is involved in the response to lipid overload in both organisms *in vivo*, while a palmitate feeding test induced ROS production, which was inhibited by antioxidant treatments in Chinese hamster ovary cell culture [144]. In studies involving human liver cells, the population with high lipid content also showed high ROS levels and metabolic dysfunction as determined through microscopy and flow cytometry [145]. In yeast engineered for elevated lipid content, high acyl-CoA or toxic fatty acid (oleic acid) accumulation was associated with increased expression of transcripts for genes that are protective to oxidative stress [146], and in separate research, these effects were accompanied by reduced cell viability (membrane integrity) [147]. Additionally, engineering cellular redox or oxidative stress defense pathways to alleviate the ROS and enhance NADPH supply was found to significantly improve yields of both standard lipid and PUFA in yeast [57,148,149].

### 3.4. Mitochondria Membrane Potential

The mitochondrial membrane potential (△ψm) generated by proton pumps from complexes III and IV is an important index for functional mitochondria [150]. Moreover, mitochondria are the major organelle to produce ROS, and mitochondrial function is found to be required for resistance to oxidative stress in the yeast *S. cerevisiae* [151]. Adaptive responses to oxidative stress may involve the opening of mitochondrial channels, such as the mitochondrial permeability transition pore and the inner membrane anion channels. As these channels get activated, this causes a change in intra- and inter-mitochondrial oxidative environment and results in the release of ROS [152]. Mitochondrial membrane potential is closely related to ROS, cell membrane integrity, and growth, which is also crucial to lipid pathway engineering. Lipotoxicity can disrupt mitochondrial respiration, either by inducing the release of cytochrome c or through interaction with mitochondrial respiratory chain complex III [142]. Flow cytometry is a useful technique to measure cellular mitochondria membrane mass or potential and employs fluorescence dyes including Rhodamine123, Rhodamine B hexyl ester, MitoTracker^®^ Green, SYTO 18, DiOC6, and TMRE.

### 3.5. Heterogeneity

Microbial life is most often described by average population behaviors, but cell-to-cell heterogeneity in the gene expression state or phenotypic heterogeneity is a widespread phenomenon and may affect the robustness of cells and result in below optimal yields in bioprocesses [153,154]. Heterogeneity among isogenic cells arises through both intrinsic (differences at all stages of metabolic processes of the cell) and extrinsic factors (external to the cell, such as oxygen and nutrient availability, temperature gradients, etc.). The understanding that heterogeneity in cell-to-cell productivity occurs within cultures and that those strategies are needed to overcome potential issues with subpopulations before transferring these cells into large-scale bioprocesses has been discussed [155,156]. For example, heterogeneity of the lipid-producing culture system, including both oil and water phases (especially for extracellular lipid production), could be an important factor leading to cell and titer heterogeneity. This should be considered when designing engineering strategies to allow cells to be more tolerant to the potential double oil and water phases in bioreactors. Heterogeneity in lipid droplet size and occurrence was indicated as a mechanism to reduce lipotoxicity in human liver cells, resulting in a population of high-lipid cells that protected not only the population of low-lipid cells, but, importantly, reduced ROS levels of the entire cell population [145]. Whether this is occurring to any degree in populations of yeast cells engineered for high lipid production is not known, nor has it been tested to date. Multiple analytical approaches, such as flow cytometry, biospectroscopy, and microfluidic single-cell cultivation, exist to assist in the understanding of microbial populations at the single cell level [157,158].

## 4. Conclusions and Outlook

This literature review summarizes the mechanisms of lipid production and current efforts of metabolic engineering to increase lipid storage (especially TAG) in yeast *S. cerevisiae*. Except for the standard lipids, it also covers the value-added FAs such as cyclopropane fatty acid, ricinoleic acid, gamma linoleic acid, EPA, and DHA. To be specific, it discusses the mechanisms of four individual and combinational major modules in lipid production, including FA biosynthesis, lipid accumulation and sequestration, and FA modification. In addition, attempts at the combination of metabolic engineering and bioprocess strategy are included. Moreover, the potential relationships between lipid pathway engineering and cellular physiological responses in cell growth, membrane integrity, intracellular reactive oxygen species, mitochondrial membrane potential, and cell populational heterogeneity are reviewed and discussed. To further engineer yeast *S. cerevisiae* for a higher production of intracellular standard or unusual lipids, it will be a challenge to raise novel metabolic engineering strategies to address the lipid droplet formation mechanism. Furthermore, intensifying and employing the understanding of cellular physiological responses (such as energy balance and ROS level) to lipid pathway modification will be a promising alternative approach to striving for a higher lipid content in engineered yeast.

## Figures and Tables

**Table 1:** Comparison of different hosts for lipid production.

Sources	Host Model	Pros	Cons	Refs
Fungi	*S. cerevisiae*	robust genetically tractable easy to culture industrial usage	low lipid yield not yet commercialized	[26,27]
Bacteria	*E. coli*	short generation time genetically tractable industrial usage	low lipid yield no stable lipid body	[7]
Microalgae	*Chlorella*	high oil productivity CO_2_ fixation	low biomass concentrations high costs for oil recovery open pond culture issue	[14,15]
Plant	*Arabidopsis* *thaliana*	high oil yield CO_2_ fixation	difficulties to industrialize deforestation greenhouse gas emission	[20]

## Data Availability

Not applicable.

## Abbreviations

ACB1, acyl-CoA binding protein; ACS, acetyl-CoA synthase; ACC, acetyl-CoA carboxylase; ADH, alcohol dehydrogenase; ACP, acyl carrier protein; CDW, cell dry weight; DAG, diacylglycerol; DGAT, acyl-CoA: diacylglycerol acyltransferase; DHAP, dihydroxyacetone phosphate; DHA, docosahexaenoic acid (22:6 ω-3); EPA, eicosapentaenoic acid (20:5 ω-3); ER, endoplasmic reticulum; FAEE(s), fatty acid ethyl ester(s); FAME, fatty acid methyl ester; FADs, fatty acid desaturases; FAS, fatty acid synthase; FFA(s), free fatty acid(s); G3P, glycerol-3-phosphate; GPAT, G3P acyltransferase; GAPN, NADP+-dependent glyceraldehyde-3-phosphate dehydrogenase; GLA, γ-linolenic acid (18:3 ω-6); GUT2, glycerol-3-phosphate dehydrogenase gene; LDs, lipid droplets; LPA, lysophophatidate; LPAT, lysophophatidate acyl-transferase; ME, malic enzyme; MCFA, medium chain fatty acid; PDAT, phospholipid: diacylglycerol acyltransferase; PA, phosphatidate; PAP, phosphatidate phosphatase; PDAT, phospholipid: diacylglycerol acyltransferase; PDCT, phosphatidylcholine diacylglycerol cholinephosphotransferase; POX, peroxisomal β-oxidation; PUFA, polyunsaturated fatty acid; tesA, encode thioesterase I; UFA, unsaturated fatty acid; WT, wild type.
